# Airway remodelling in asthma and the epithelium: on the edge of a new era

**DOI:** 10.1183/13993003.01619-2023

**Published:** 2024-04-18

**Authors:** Gilda Varricchi, Christopher E. Brightling, Christopher Grainge, Bart N. Lambrecht, Pascal Chanez

**Affiliations:** 1 Department of Translational Medical Sciences and Center for Basic and Clinical Immunology Research (CISI), School of Medicine, University of Naples Federico II, WAO Center of Excellence, Naples, Italy; 2 Institute of Experimental Endocrinology and Oncology (IEOS), National Research Council, Naples, Italy; 3 Institute for Lung Health, NIHR Leicester Biomedical Research Centre, University of Leicester, Leicester, UK; 4 School of Medicine and Public Health, University of Newcastle, Callaghan, Australia; 5 Center for Inflammation Research, Laboratory of Immunoregulation and Mucosal Immunology, VIB Center for Inflammation Research, Ghent, Belgium; 6 Department of Respiratory Diseases, Aix-Marseille University, Marseille, France; 7 G. Varricchi and C.E. Brightling contributed equally

## Abstract

Asthma is a chronic, heterogeneous disease of the airways, often characterised by structural changes known collectively as airway remodelling. In response to environmental insults, including pathogens, allergens and pollutants, the epithelium can initiate remodelling *via* an inflammatory cascade involving a variety of mediators that have downstream effects on both structural and immune cells. These mediators include the epithelial cytokines thymic stromal lymphopoietin, interleukin (IL)-33 and IL-25, which facilitate airway remodelling through cross-talk between epithelial cells and fibroblasts, and between mast cells and airway smooth muscle cells, as well as through signalling with immune cells such as macrophages. The epithelium can also initiate airway remodelling independently of inflammation in response to the mechanical stress present during bronchoconstriction. Furthermore, genetic and epigenetic alterations to epithelial components are believed to influence remodelling. Here, we review recent advances in our understanding of the roles of the epithelium and epithelial cytokines in driving airway remodelling, facilitated by developments in genetic sequencing and imaging techniques. We also explore how new and existing therapeutics that target the epithelium and epithelial cytokines could modify airway remodelling.

## Shareable abstract

**Our growing understanding of how the epithelium and epithelial cytokines orchestrate airway remodelling in severe asthma is enabling the development of new therapies that may make disease remission a realistic treatment goal** https://bit.ly/3UOblpT

## Introduction

Asthma is a chronic, inflammatory disease of the airways, characterised by variable symptoms of wheezing, chest tightness, cough, shortness of breath and varying expiratory airflow limitation [1]. The disease is phenotypically heterogeneous, with differing clinical characteristics such as age at onset, degree of severity and response to treatment [2]. Heterogeneity in the biological and immunological mechanisms that contribute to asthma led to the conception of “endotypes” to define groups of patients with similar disease characteristics at the cellular and molecular levels, such as features of inflammation [3]. The most common inflammatory endotype is type 2 (T2)-high asthma, characterised by high levels of T2 inflammatory biomarkers including eosinophils, exhaled nitric oxide, IgE, interleukin (IL)-5 and IL-13 [4]. Patients with T2-low asthma exhibit lower levels of T2 inflammation; instead, the dominant inflammatory cell types may include neutrophils or there might be very few inflammatory cells, termed paucigranulocytic inflammation [5, 6]. Neutrophilic inflammation can coexist with eosinophilic inflammation in patients with severe asthma with a mixed phenotype [5, 7]. Endotypes and phenotypes are dynamic, and diverse environmental stimuli may induce modifications resulting in mixed molecular pathways and phenotypes that overlap over a patient's lifetime [8].

The airway epithelium plays a critical role in the pathophysiology of asthma, acting as a structural and immunological barrier to the external environment [9]. Airborne pathogens, pollutants and, in sensitised people, allergens can damage airway epithelial cells and trigger the release of epithelial cytokines that drive downstream inflammatory processes [10–12]. In the healthy state, resolution of acute inflammation can repair and restore normal airway structure and function, avoiding epithelial damage. However, in patients with asthma, aberrant immune responses and repair processes lead to recurrent or chronic inflammation and damage to the airway epithelium, which can result in structural changes in the large and small airways [13]. These structural changes, collectively referred to as airway remodelling, include epithelial dysfunction, goblet cell hyperplasia and metaplasia, thickening and fibrosis of the subepithelial matrix, increased airway smooth muscle (ASM) mass and enhanced angiogenesis. These features contribute to narrowing and stiffening of the airways, resulting clinically in airflow limitations with subsequent worsening of respiratory symptoms [14].

Airway remodelling is a near-universal feature of asthma. It is present in patients with mild disease [15] but tends to worsen with increasing disease severity [16, 17], and is associated with a higher exacerbation risk, lung function decline and disease chronicity [9, 14, 18]. Airway remodelling was once considered a secondary phenomenon, developing in late-stage asthma as a consequence of chronic inflammation. However, biopsy studies in young children indicate that airway remodelling can be an early event in asthma development, initiating before symptoms occur [19–21] and potentially even predisposing individuals to developing asthma by affecting lung development [9]. Structural changes to the airways are believed to persist through adulthood [22].

Developments in genetic sequencing and imaging techniques have advanced our understanding of the roles of the epithelium and epithelial cytokines in driving airway remodelling, taking us to the edge of a new era of scientific discovery and potential therapeutic development. Here, we review the emerging data in this growing area of research, their clinical relevance, and how new and existing therapeutics that target the epithelium and epithelial cytokines could modify airway remodelling in asthma.

## How the epithelium orchestrates airway remodelling

The epithelial cells of the airways act as an initiation point for airway remodelling in asthma [14]. Epithelial cells express pattern recognition receptors, which detect pathogen-associated molecular patterns and damage-associated molecular patterns derived from pathogens, allergens and injured cells resulting from environmental insults such as pollutants and toxins [23]. The triggering of pattern recognition receptors results in the epithelial release of chemokines and cytokines (primarily epithelial cytokines and ILs) and a downstream inflammatory cascade involving various immune and structural cells [23–25]. Pro-inflammatory stimuli and cytokines also trigger epithelial inducible nitric oxide synthase expression, producing an increase in nitric oxide levels. This induces chronic inflammatory responses and nitration of proteins involved in proliferation, apoptosis or migration, triggering epithelial tissue injury [26]. Environmental insults may also induce apoptosis of the epithelium, accompanied by the release of mediators such as transforming growth factor (TGF)-β [27], which can initiate the tissue regeneration process in an attempt to restore homeostasis. However, persistent inflammation and damage to the epithelium can lead to aberrant tissue repair and pathological remodelling of the airways [9, 28, 29].

Multiple mechanical forces, including compression (cells being pushed together), stretch (of cells at the apex of the epithelial folds) and shear stress (increase in air velocity together with a reduction in airway diameter), affect the epithelium as the airway wall folds [30, 31]. A variety of experimental models have been used to show that mechanical forces can initiate airway remodelling independently of inflammation [32]. The mechanical forces present during regular respiration are balanced; however, during bronchoconstriction experienced by patients with asthma, airway epithelial cells are exposed to excessive mechanical forces [14]. This compressive stress results in mechanical stimulation, which elicits a pro-remodelling response in the absence of inflammation [33, 34]. This response causes subepithelial thickening *via* increased production of fibronectin and collagen III and V, and an increase in the ratio of matrix metalloproteinase (MMP)-9 to tissue inhibitor of metalloproteinase-1 [33]. Goblet cell hyperplasia and metaplasia, and subsequent mucus overproduction, may also occur as a result of epithelial compression during bronchoconstriction [34].

Airway remodelling may be self-perpetuating. Epithelial disruption can result in an abnormal epithelium-mediated immune response, potentially promoting the persistence of microbes such as bacteria and fungi in the airways [35]. Meanwhile, stiffening of the airway walls alters the local biomechanical environment. Both of these abnormalities can drive a positive feedback loop that perpetuates airway remodelling [14, 36].

## Role of epithelial cytokines in airway remodelling

Three epithelial cytokines, *i.e.* thymic stromal lymphopoietin (TSLP), IL-33 and IL-25, known as “alarmins”, are central in asthma pathophysiology. They act as master regulators that mediate both innate and adaptive immune responses, including both T2 and non-T2 inflammation, as well as structural changes in the airways [25, 37]. Airway levels of both TSLP and IL-33 are elevated in patients with asthma compared with healthy individuals and correlate with disease severity [38–42]. IL-25 concentrations in sputum may also correlate with disease severity [43].

**FIGURE 1:**
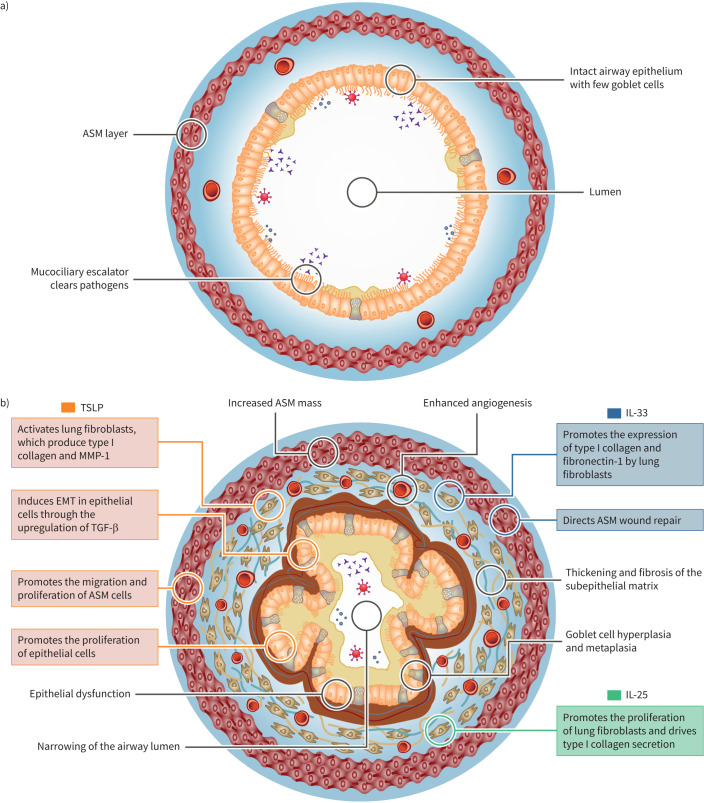
Multiple pathogenic factors trigger structural alterations of a) healthy airways resulting in b) airway remodelling. Epithelial cytokines can play diverse, yet often overlapping, roles in airway remodelling in asthma. ASM: airway smooth muscle; EMT: epithelial-to-mesenchymal transition; IL: interleukin; MMP: matrix metalloproteinase; TGF: transforming growth factor; TSLP: thymic stromal lymphopoietin.

The three epithelial cytokines have diverse, yet often overlapping, effects on mesenchymal cells such as lung fibroblasts and ASM cells (figure 1). TSLP activates lung fibroblasts, which produce extracellular matrix (ECM) molecules such as collagen I and MMP-1 [44–46]. There is also evidence that human endothelial cells express the TSLP receptor and that TSLP induces their proliferation [47, 48]. Moreover, TSLP promotes the release of vascular endothelial growth factor (VEGF)-A from human lung macrophages [49]. IL-33 promotes the expression of collagen and fibronectin-1 in lung fibroblasts [50, 51], and IL-25 promotes lung fibroblast proliferation [52] and collagen secretion by these cells [53]. In turn, asthmatic lung fibroblasts contribute to inflammation by secreting cytokines, including TSLP and IL-33 [54, 55].

During epithelial-to-mesenchymal transition (EMT), epithelial cells lose their epithelial markers, migrate to the lamina propria and gain mesenchymal markers. These mesenchymal cells now synthesise ECM, which provides a framework for basal cells to replace damaged epithelium in wound healing [56]. *In vitro* studies have shown that TSLP- and IL-33-mediated signalling between the epithelium and fibroblast-like mesenchymal cells may drive remodelling in response to recurring injury, potentially (for TSLP) through upregulating the expression of TGF-β [14, 57, 58]. However, there is a lack of *in vivo* evidence regarding the role of EMT in the pathogenesis of asthma.

Both TSLP and IL-33 mediate cross-talk between ASM and mast cells, with subsequent effects on airway structure and function [40, 59]. TSLP promotes the proliferation and migration of ASM cells and the release of inflammatory cytokines, including TSLP itself, from these cells [60–62]. TSLP also acts upon mast cells, which in turn can trigger bronchoconstriction and increased ASM mass [63]. IL-33 directs ASM wound repair and drives airway hyperresponsiveness (AHR) through IL-13-based signalling between ASM and mast cells [40]. Mast cells play a key role in asthma pathophysiology and airway remodelling through the release of a plethora of cytokines (*e.g.* TSLP, IL-33, IL-25, IL-4, IL-13 and TGF-β) and angiogenic factors (*e.g.* VEGF-A), as well as other inflammatory mediators and bronchoconstrictors [64, 65]. IL-25 contributes to airway remodelling *via* the induction of airway angiogenesis in murine asthma models [53, 66].

## Downstream immune cell actions influence airway remodelling

**FIGURE 2:**
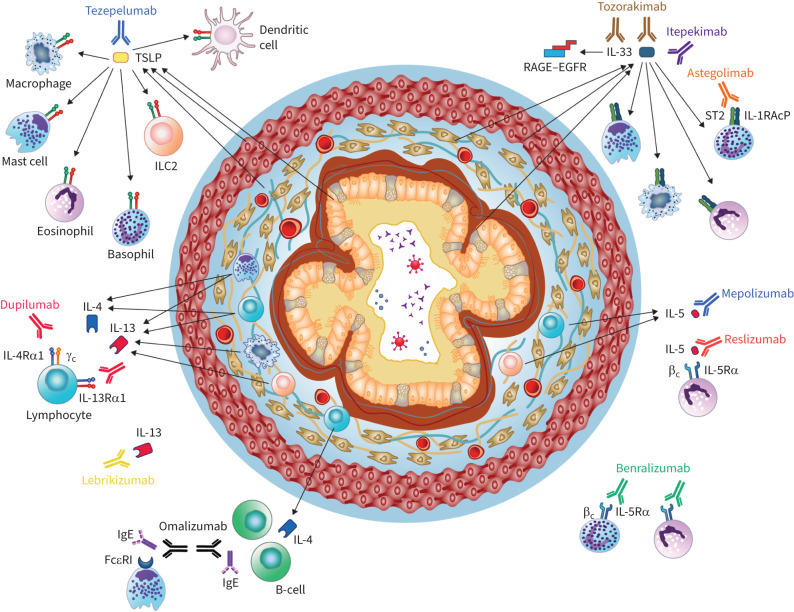
Epithelial cytokines activate immune cells that drive pathogenic airway remodelling processes in patients with severe asthma. Biologic therapies for severe asthma (approved or recently in development) that target epithelial cytokines and downstream mediators may ameliorate features of airway remodelling. EGFR: epidermal growth factor receptor; IL: interleukin; ILC2: group 2 innate lymphoid cell; R: receptor; RAGE: receptor for advanced glycation end-products; ST2: serum stimulation-2; TSLP: thymic stromal lymphopoietin.

Epithelial cytokines activate several immune cells that can drive airway remodelling, including dendritic cells, T-helper type 2 (Th2) cells, group 2 innate lymphoid cells (ILC2s), eosinophils and macrophages (figure 2) [67, 68].

During T2 inflammatory responses, TSLP activates dendritic cells, which in turn prime naive T-helper cells to produce Th2-like cytokines such as IL-4, IL-5 and IL-13 [69]. IL-33 acts as a positive regulator of TSLP–dendritic cell signalling, initiating and maintaining Th2 cell-mediated inflammatory responses [70]. Moreover, TSLP, IL-33 and IL-25 activate ILC2s, which also produce IL-4, IL-5 and IL-13 [71–73]. The IL-5 secreted from polarised Th2 cells and ILC2s stimulates eosinophilic inflammation *via* effects on eosinophil recruitment, maturation and survival [74]. Activated eosinophils and mast cells release cysteinyl leukotrienes, which are potent bronchoconstrictors and induce airway remodelling through ASM cell proliferation and the release of mediators such as TGF-β, cationic proteins and cytokines [75, 76]. Both the *MET* gene, encoding the hepatocyte growth factor receptor, and the *MMP10* gene, encoding MMP-10, are implicated in airway remodelling and cellular inflammation associated with submucosal eosinophils [77]. IL-4 and IL-13 enhance subepithelial fibrosis, mucus production *via* goblet cell proliferation and collagen deposition [14].

TSLP may also play a role in non-T2 inflammation-mediated airway remodelling. Dendritic cells activated by TSLP can induce the polarisation of naive T-cells towards a T-helper type 17 (Th17) phenotype [78]. The synergistic effect of dendritic cells together with Th17 cytokines promotes neutrophilic inflammation and accumulation of fibrotic matrix components that correlate with TGF-β expression [79, 80].

All three epithelial cytokines can activate human lung macrophages [25, 49]. Macrophages influence inflammatory responses in the airways through phagocytosis, cytokine and angiogenic factor production, and regulation of tissue repair in the lungs [81, 82]. Alveolar macrophages are activated by TGF-β and release MMPs that alter the ECM and airway structure [14, 49]. Two distinct populations of resident macrophages have been identified in the lungs [83–85]. One macrophage population (Lyve1^lo^MHCII^hi^) is mostly located adjacent to nerves and primarily presents antigens. The other (Lyve1^hi^MHCII^lo^) resides alongside blood vessels; in a mouse model, Lyve1^hi^MHCII^lo^ macrophages were shown to have a critical role in suppressing inflammation and fibrosis [83–85].

## Influence of genetic and epigenetic changes in the airway epithelium on remodelling

Genetic variations in the airway epithelium can initiate or worsen asthma and influence remodelling [86–88]. In the largest genome-wide association study to date in moderate-to-severe asthma, Shrine *et al.* [89] reported that variants in *MUC5AC*, *GATA3* and *KIAA1109* were associated with an increased susceptibility to developing moderate-to-severe asthma. *MUC5AC* gene variants are associated with increased mucus plugging, whereas *GATA3* is a transcription factor regulating T2 immunity and allergy. The function of *KIAA1109* is not fully understood [89].

Heijink *et al.* [90] selected 12 genes identified in asthma genetic studies that are implicated in epithelial function. These include genes related to the inflammatory environment (*IL33*, *TSLP* and *IL1RL1*), the response to pathogens (*CDHR3*), mucociliary clearance (*MUC5AC*, *KIF3A* and *EFHC1*), and cell homeostasis and epithelial integrity, including proliferation, migration, cell–cell adhesion, apoptosis and repair (*PCDH1*, *SMAD3*, *GSDMB*, *ORMDL3* and *PLAUR*). Relevant to the latter group of genes, the 17q21 gene locus, which is linked to *GSDMB* and *ORMDL3*, is an important susceptibility locus for childhood-onset asthma [91]. Gasdermin B (GSDMB) is highly expressed in the epithelium in asthma, and increased GSDMB expression in transgenic mice leads to spontaneous remodelling and AHR in the absence of airway inflammation [92]. Orosomucoid-like protein isoform 3 (ORMDL3) is expressed in epithelial cells and regulates endoplasmic reticulum stress and sphingolipid homeostasis [93]. Transgenic mice overexpressing ORMDL3 exhibit increased ASM mass, subepithelial fibrosis and mucus [94]. Plasminogen activator urokinase receptor (PLAUR) is expressed in the epithelium and regulates the activation of urokinase plasminogen activator, triggering the plasminogen/plasmin activation cycle. PLAUR is also involved in epithelial repair, proliferation and remodelling [95]. In patients with asthma, *PLAUR* variants are associated with a rapid decline in lung function and with airway remodelling through effects on reticular basement membrane (RBM) thickness, collagen III deposition and basal epithelial proliferation [96].

Lastly, although its function is unclear, elevated serum levels of YKL-40, a chitin-binding glycoprotein expressed in the airway epithelium, correlate with asthma severity, airway remodelling and increased thickness of the RBM [97]. Variations in the expression of the *CHI3L1* gene, which encodes YKL-40, are also associated with asthma severity, airflow obstruction and airway remodelling [98].

Epigenetic changes, such as DNA methylation or acetylation, histone modifications and microRNA modifications, in the airway epithelium are associated with asthma [10, 12, 99, 100]. The DNA methylation pattern of epithelial cells is different for children with asthma compared with healthy children or those with atopic asthma [101]. School-aged children with asthma also have differential methylation of genes relevant to epithelial barrier function, airway epithelial integrity and immune regulation [102]. Of note, overexpression of TSLP in asthmatic airway epithelial cells may be regulated by DNA demethylation [103]. Increased histone deacetylase activity could be responsible for tight junction dysfunction in asthma, and inhibiting this activity is a promising target for improved barrier integrity [104, 105]. Lastly, epigenetic “training” from repetitive activation of innate signalling pathways can induce adaptive epithelial responses [106]. Oxidative stress resulting from the innate immune response can cause conformational changes in DNA that trigger inflammation, EMT and ECM remodelling. Targeting inducible epigenetic guanine oxidation reprogramming pathways reduced airway inflammation in pre-clinical models [106].

## Structural and clinical consequences of airway remodelling

The ultrastructural changes associated with airway remodelling result in altered airway geometry, including airway wall thickening and luminal narrowing, and in ventilation defects, including mucus plugging and airway obstruction. Some of these phenomena can be assessed using imaging techniques such as computed tomography (CT) [107, 108].

Goblet cell hyperplasia and submucosal gland hypertrophy lead to increased sputum production, airway narrowing due to sputum secretion and increased airway wall thickness [109]. These changes can ultimately result in the formation of mucus plugs, which are associated with severe airflow limitation and death [110, 111]. Subepithelial fibrosis resulting from increased ECM deposition leads to airway wall thickening, which correlates with asthma severity and AHR [109, 112, 113]. Increased ASM mass is associated with asthma severity [114], and ASM cells migrating towards the epithelium and subsequent increased ECM deposition within the ASM may contribute to airflow obstruction [109, 115]. Changes in airway wall microvasculature resulting from inflammatory angiogenesis can contribute to the development of airway wall oedema, leading to luminal narrowing [109]. Lastly, decreased cartilage volume and increased cartilage proteoglycan degradation in the airways can contribute to chronic airway obstruction and enable more powerful bronchoconstriction for a given degree of ASM contraction [109].

Although the qualitative assessment of chest radiographs and CT scans is part of the standard of care for asthma, other lung imaging platforms and software algorithms can quantify regional airway structure and function, as well as airway inflammation. In addition to CT imaging, new approaches such as hyperpolarised gas magnetic resonance imaging and single-photon emission CT are helping us to understand the heterogeneity of severe asthma and are likely to advance clinical management towards precision medicine [116].

Measuring the geometry of the airways provides a measure of the impact of remodelling at an individual airway level, whereas lung function tests measure remodelling at an organ level. However, the ultrastructural/cellular changes that lead to remodelling do not happen on the same timescale as the development of clinical symptoms (*e.g.* breathlessness). Access to and combining information from different modalities will provide insight into the mechanisms underlying airway remodelling [35].

## Identification of airway remodelling phenotypes in asthma

Bronchial biopsy and post-mortem tissue section studies show differences in airway structural characteristics between patients with different asthma phenotypes. Patients with severe asthma and high levels of eosinophilic inflammation have greater RBM thickness than those with nearly absent eosinophils [117]. In a subsequent study, no difference in RBM thickness was found between patients with early-onset (childhood) and late-onset (adulthood) asthma, despite those with adult-onset asthma having inferior forced expiratory volume in 1 s (FEV_1_) and forced vital capacity and a shorter duration of disease [118]. Among patients with late-onset asthma, those with eosinophilic inflammation had greater RBM thickening than those without eosinophilic inflammation. Patients with paucigranulocytic asthma had increased thicknesses of the ASM layer and RBM compared with healthy individuals [119]. Meanwhile, patients with granulocytic asthma also had increased airway wall thickness and narrowing of the airway lumen due to ASM shortening and mucus obstruction [119]. This finding suggested that some components of remodelling are dependent on inflammation whereas others are not. In the paucigranulocytic endotype, airway remodelling is thought to occur in a manner “uncoupled” from inflammation, potentially through mechanotransduction-related pathways [6].

Attempts have also been made to identify airway remodelling phenotypes using quantitative CT imaging [120, 121]. Cluster analysis of wall and lumen volumes of the large airways in an adult asthma cohort identified three phenotypes, all of which demonstrated air trapping: one cluster with increased airway wall and lumen volumes and another cluster with luminal narrowing, both with poor lung function, and a third cluster lacking airway remodelling that had clinically mild disease [121]. A second analysis of remodelling across the small and large airways in a cohort of patients with severe asthma identified three phenotypes: one characterised by large-to-medium bronchial wall thickening, mucus plugging and bronchiectasis, associated with systemic eosinophilic inflammation; one characterised by small airway remodelling and fixed airflow obstruction, associated with male sex, smoking and more frequent use of controller medication; and one lacking both eosinophilic inflammation and airway remodelling [120].

Successful identification of remodelling phenotypes may enable the development of targeted approaches to treat traits specifically related to airway remodelling, with the aim of preventing lung function decline. The combination of clinical, inflammatory and remodelling phenotypes within a patient could determine the optimal personalised approach to their treatment [22].

## Targeting the epithelium and epithelial cytokines to modify airway remodelling

Various therapies for asthma target the epithelium either directly or indirectly and, as well as improving clinical symptoms, may ameliorate features of airway remodelling and lung function decline as measured by FEV_1_ and other spirometric parameters.

### Glucocorticoids

First-line therapy for asthma typically includes inhaled corticosteroids (ICS), targeting glucocorticoid receptors that are expressed almost ubiquitously, including in epithelial cells. Maintenance use of ICS (≥1 year) is associated with modest improvements in FEV_1_ [122]. Glucocorticoids mitigate chronic inflammation, which indirectly contributes to airway remodelling. However, evidence for a direct beneficial impact of ICS or oral corticosteroids on airway remodelling has been contradictory. High-dose ICS reduced both submucosal vascularity and RBM thickness in patients with mild-to-moderate asthma [64]. Glucocorticoid treatment also restored the integrity of epithelial cell monolayers through the redistribution of tight junction proteins [123]. Furthermore, studies in epithelial cells suggest that glucocorticoid exposure may reduce goblet cell hyperplasia [124, 125], but ICS treatment in patients with mild asthma did not confirm this [126]. Glucocorticoid treatment may in fact contribute to airway remodelling, potentially by inducing caspase-mediated epithelial cell apoptosis [127].

### Biologics targeting T2 inflammation

Biologic therapies may be prescribed as add-on maintenance treatments to improve disease control in patients with moderate-to-severe asthma [1]. Monoclonal antibodies (mAbs) that target IgE or Th2 cytokines (IL-5, IL-4 and IL-13) and their receptors have secondary effects on the epithelium through their actions on these immune cell mediators (figure 2 and table 1) [128, 129]. The possible effects of these biologics on airway remodelling have been reviewed in detail elsewhere [128–130] and are summarised briefly here.

**TABLE 1:** Effects of biologics for severe asthma on airway remodelling and clinical outcomes [129, 130]

**Biologic**	**Target**	**GINA eligibility criteria (in addition to having severe asthma)**	**Biological effects**	**Effects on airway remodelling**	**Expected clinical outcomes**
**Omalizumab**	IgE	Severe exacerbations within past year, sensitisation to inhaled allergens, total serum IgE and weight within local dosing range	↓ Circulating total IgE Downregulation of FcεRI receptors on basophils, mast cells and dendritic cells	↓ RBM thickness ↓ Airway wall thickness on CT ↓ Fibronectin deposition Prevents IgE-mediated ECM deposition *in vitro*	↑ Lung function (FEV_1_) ↓ Severe exacerbations ↑ Health-related QoL ↑ Symptom control ↓ OCS (possible benefit)
**Mepolizumab**	IL-5	Severe exacerbations within past year, BEC ≥150 or ≥300 cells·µL^−1^ (locally specified)	Blockage of IL-5/IL-5R binding	↓ Airway eosinophils and TGF-β1^+^ eosinophils ↓ Tenascin, lumican and procollagen III expression ↓ RBM thickness ↓ Airway wall thickness on CT ↓ ASM mass	↑ Lung function (FEV_1_) ↓↓ Severe exacerbations ↑ QoL ↑ Symptom control ↓ OCS
**Reslizumab**	IL-5	Severe exacerbations within past year, BEC ≥150 or ≥300 cells·µL^−1^ (locally specified)	Blockage of IL-5/IL-5R binding	Not reported	↑ Lung function (FEV_1_) ↓↓ Severe exacerbations ↑ QoL ↑ Symptom control
**Benralizumab**	IL-5Rα	Severe exacerbations within past year, BEC ≥150 or ≥300 cells·µL^−1^ (locally specified)	↓ Eosinophils and basophils *via* antibody-dependent cell-mediated cytotoxicity	↓ Airway eosinophils ↓ ASM mass	↑ Lung function (FEV_1_) ↓↓ Severe exacerbations ↑ Health-related QoL ↑ Symptom control ↓ OCS
**Dupilumab**	IL-4Rα	Severe exacerbations within past year, BEC ≥150 and ≤1500 cells·µL^−1^, or *F*_ENO_ ≥25 ppb, or maintenance OCS	Blockage of IL-4/IL-4Rα binding Blockage of IL-13/IL-4Rα binding	Prevents eosinophil infiltration into lung tissue in a mouse model of asthma	↑ Lung function (FEV_1_) ↓↓ Severe exacerbations ↑ Health-related QoL ↑ Symptom control ↓ OCS
**Tezepelumab**	TSLP	Severe exacerbations within past year	Blockage of TSLP/TSLPR binding	↓ Airway eosinophils ↓ AHR to mannitol ↓ Airway inflammation ↓ TGF-β1 ↑ CT scan-determined lumen area	↑ Lung function (FEV_1_) ↓↓ Severe exacerbations ↑ Health-related QoL ↑ Symptom control ↓ OCS (possible benefit)

AHR: airway hyperresponsiveness; ASM: airway smooth muscle; BEC: blood eosinophil count; CT: computed tomography; ECM: extracellular matrix; *F*_ENO_: fractional exhaled nitric oxide; FEV_1_: forced expiratory volume in 1 s; GINA: Global Initiative for Asthma; IL: interleukin; OCS: oral corticosteroids; QoL: quality of life; R: receptor; RBM: reticular basement membrane; TGF: transforming growth factor; TSLP: thymic stromal lymphopoietin.

Omalizumab binds to free IgE, inhibiting its binding to the high-affinity IgE receptor FcεRI on mast cells, basophils and dendritic cells [131]. Although omalizumab did not improve FEV_1_ in randomised controlled trials (RCTs) [132, 133], there is some evidence for FEV_1_ improvement and reduction in severe exacerbations with omalizumab in real-world settings [134, 135]. Omalizumab reduced RBM thickness [136, 137] and fibronectin deposits in asthmatic airways [138] and prevented ASM remodelling *in vitro* [139].

Mepolizumab and reslizumab bind to IL-5, preventing it from binding to the IL-5 receptor α subunit (IL-5Rα) on eosinophils, thereby reducing eosinophil activation and maturation [140, 141]. Benralizumab has a similar mechanism of action, targeting IL-5Rα [142]. These biologics improved FEV_1_ in RCTs in patients with severe eosinophilic asthma [143–147] and in real-world settings [148–150]. Mepolizumab treatment reduced levels of the ECM proteins tenascin, lumican and procollagen III and airway eosinophils expressing TGF-β1 in the bronchial biopsies of patients with mild atopic asthma, as well as TGF-β1 levels in bronchoalveolar lavage [151]. In patients with refractory eosinophilic asthma, a reduction in CT-measured airway wall area was observed with mepolizumab [152]. Preliminary results from the MESILICO study showed that mepolizumab significantly reduced basement membrane thickness, ASM area, the extent of epithelial damage and tissue eosinophil numbers in patients with late-onset, severe eosinophilic asthma and fixed airflow obstruction [153]. Benralizumab reduced both eosinophil numbers in the bronchial lamina propria and ASM mass in patients with severe eosinophilic asthma, with no significant change in myofibroblast numbers. The effects of benralizumab on ASM mass were attributed to an indirect effect mediated by the depletion of TGF-β1^+^ eosinophils [154]. The effects of reslizumab on airway remodelling have not yet been reported.

Dupilumab binds to the IL-4 receptor α subunit found on lymphocytes, including B- and T-cells, as well as on epithelial cells, blocking both IL-4 and IL-13 signalling [155]. This may be expected to reduce mucus production, goblet cell hyperplasia, subepithelial fibrosis and collagen deposition [14, 156]. In patients with severe asthma, dupilumab improved FEV_1_ and other lung function measures both in RCTs [157, 158] and a real-world study [159]. An ongoing RCT is evaluating the effects of dupilumab on lung function and airway remodelling using functional respiratory imaging, a novel technology that uses high-resolution CT scans to quantify airway structure and function [160].

Although not currently an approved therapy for asthma, treatment with the anti-IL-13 mAb lebrikizumab reduced subepithelial collagen thickness in patients with uncontrolled asthma, providing further evidence of a role for IL-13 in airway remodelling [161].

In a proof-of-concept study, combined vaccination against IL-4 and IL-13 showed both prophylactic and therapeutic efficacy in a mouse model of asthma [162]. This opens the path for clinical development of a vaccine against asthma that would be a cost-effective alternative to therapeutic antibodies.

### Biologics targeting epithelial cytokines

Targeting the epithelium more directly by inhibiting the epithelial cytokines TSLP, IL-33 and IL-25 may be a promising approach to improving airway remodelling (figure 2). Tezepelumab, a human mAb that binds TSLP, has recently been approved for the treatment of patients with severe asthma, with no phenotype or biomarker limitations (table 1) [163]. In phase 2 and 3 RCTs in patients with severe, uncontrolled asthma, there were rapid and sustained improvements in FEV_1_ and other lung function measures with tezepelumab compared with placebo [164, 165]. Tezepelumab also reduced serum levels of MMP-3 and MMP-10 [166]. In an exploratory, mechanistic study in patients with moderate-to-severe asthma, tezepelumab significantly reduced submucosal eosinophil counts in bronchial biopsies compared with placebo, irrespective of baseline blood eosinophil count [167]. There were no significant differences between treatment groups in RBM thickness and epithelial integrity, although there were greater increases in the CT-measured lumen area across airway generations with tezepelumab than with placebo [167]. The increases in lumen area were potentially related to reductions in occlusive mucus plugs, which correlated with improvements in FEV_1_ and eosinophilic inflammation [168]; this was the first demonstration that an asthma biologic could reduce mucus plugs. AHR to mannitol was also reduced with tezepelumab compared with placebo in CASCADE [167], a finding confirmed in an independent study [169], indicating that tezepelumab has effects independent of T2 inflammation. Further evidence for the benefits of TSLP blockade on remodelling comes from the use of anti-TSLP antibodies in animal studies. Blocking TSLP reduced airway inflammation and hyperresponsiveness, together with TGF-β1 levels and airway remodelling, in a mouse model of allergic asthma [170] and attenuated airway inflammation and remodelling in asthmatic rats [171]. Furthermore, TSLP blockade suppressed airway remodelling in a mouse model of asthma *via* reduced MMP, TGF-β and connective tissue growth factor levels [172].

Several biologic therapies targeting IL-33 signalling are, or have recently been, in development for asthma [173, 174]. Itepekimab is an anti-IL-33 mAb that improved FEV_1_ compared with placebo after 12 weeks of treatment in patients with moderate-to-severe asthma [173]. By contrast, astegolimab, a human mAb that inhibits the IL-33 receptor serum stimulation-2 (ST2), did not significantly improve FEV_1_ after 54 weeks of treatment in patients with severe asthma, although exacerbations were reduced [174]. However, neither itepekimab nor astegolimab are currently being pursued in asthma [175, 176]. Lastly, tozorakimab is a novel dual-pharmacology mAb that inhibits IL-33 activity *via* both the ST2 and receptor for advanced glycation end-products–epidermal growth factor receptor complex signalling pathways [177]. Tozorakimab reduced biomarkers of inflammation, including serum IL-5, IL-13 and blood eosinophils, in a phase 1 study [178] and is being evaluated in a phase 2 study in patients with asthma (ClinicalTrials.gov: NCT04570657). Although the impact of these mAbs on airway remodelling has not yet been assessed in clinical studies, IL-33 blockade prevented exacerbations in a mouse model of chronic airway inflammation by blunting persistent inflammation and remodelling [179]. Furthermore, in a mouse model of asthma, ST2 knockout reduced airway inflammation, Th2 cytokine expression and fibrosis-related protein deposition, all of which were further reduced by additional blockade with anti-TSLP and anti-IL-25 antibodies [180].

No biologics targeting IL-25 or its receptor are approved or known to be in late-stage development for the treatment of asthma. However, in a mouse model of allergen-induced airway remodelling, IL-25 blockade reduced airway eosinophils and levels of Th2 cytokines, and abrogated peribronchial collagen deposition, ASM hyperplasia and airway hyperreactivity [53].

## Future perspectives

Airway remodelling is a composite term used to describe the changes observed at the cell-to-tissue level within the airways of patients with asthma. Although imaging and physiology are highly predictive of the presence of remodelling, they do not provide insight into the underlying mechanisms. Our understanding of how airway remodelling mechanisms impact asthma development is limited by the lack of consensus on which airway remodelling parameters to study and a lack of appropriate tissue samples or longitudinal sampling approaches for this slowly progressing process [181]. Other fundamental limitations in airway remodelling research include a lack of relevant model systems and difficulty in accurately measuring critical indices (including biomarkers) that encompass genetic, molecular, biochemical, anatomical and functional aspects of airway remodelling. Research is also limited by the costs of the sensitive and specific techniques necessary to identify and quantify longitudinal changes in airway remodelling [181].

Multidisciplinary efforts are needed to identify accessible and valid biomarkers of airway remodelling induction, maintenance, progression and responsiveness to therapy [181, 182]. The most relevant biomarkers are likely to be those linked to pathways that can be successfully targeted by interventions and have consequent benefits at an organ level, with associated improvements in asthma control and exacerbations. Integrating data from multiple disease aspects (structural and inflammatory cell interactions, genetic background, environmental exposure to allergens, pollutants and pathogens, as well as omics) can also be used to develop statistical models of the future risk of developing frequent exacerbations or lung function decline, or the likelihood of responding to therapy [35].

To date, asthma therapies have shown limited effects on airway remodelling [181]. However, the effects of newer therapies, such as those directed against TSLP and IL-33, are yet to be fully elucidated. Nevertheless, it is important to improve the targeting of biological treatments to the most suitable patients. The 3TR (Taxonomy, Treatment, Target and Remission) consortium, involving European academic researchers, patients and the pharmaceutical industry, has set out a plan to increase the clinical impact of targeted immune-mediated therapies for asthma and other immune diseases [183]. Their key aim is to define outcome measures for documenting the therapeutic response to enable comparisons across different studies. The 3TR consortium will then study biomarkers and immunological mechanisms related to different responder profiles and will use advanced omics and systems biology analyses to identify biomarkers for predicting response. Their goal is to uncover future treatment targets and to move towards more ambitious treatment goals for patients with asthma using precision medicine [183].

## Conclusion

Evidence shows that airway remodelling can result from the combination of two major components. The inflammatory component is associated with persistent airway infiltration and activation of a wide spectrum of cells of the innate and adaptive immune systems [88, 129]. The structural component is characterised by changes involving goblet cell metaplasia, ASM hypertrophy/hyperplasia, RBM thickening, increased sensory nerve endings and increased angiogenesis [64, 129, 184, 185]. The combination of inflammatory and structural changes in airway remodelling provides the basis of what is canonically referred to as fixed airway obstruction. T2 inflammation, involving eosinophils, mast cells, ILC2s, Th2 cells and basophils, contributes to airway remodelling in most patients with severe asthma [128, 186, 187]. The contribution of non-T2 inflammation to airway remodelling, presumably involving mast cells, macrophages and neutrophils [49, 63], remains to be fully clarified [186, 188, 189].

Increasing evidence indicates that biological therapies targeting IgE, IL-5/IL-5Rα, TSLP and IL-33/ST2 can improve not only clinical symptoms but also certain features of airway remodelling in asthma [128, 129]. With continuing development of new therapies, the achievement of asthma remission and the prevention of airway remodelling could become realistic possibilities in the treatment of severe asthma.

## Shareable PDF

10.1183/13993003.01619-2023.Shareable1 This one-page PDF can be shared freely online.
Shareable PDF ERJ-01619-2023.Shareable
