# Supplementary material for: Airway remodelling in asthma and the epithelium: on the edge of a new era
Source: Eur Respir J. 2024 Apr 18;63(4):2301619. doi: 10.1183/13993003.01619-2023 (PMC11024394; doi:10.1183/13993003.01619-2023)
Supplement: Supplementary file 1 [file ERJ-01619-2023.Shareable.pdf]

EUROPEAN RESPIRATORY JOURNAL
REVIEW
G. VARRICCHI ET AL.

SHAREABLE PDF

Airway remodelling in asthma and the epithelium: on the edge of a new era

Gilda Varricchi[1,2,7], Christopher E. Brightling[3,7], Christopher Grainge[4], Bart N. Lambrecht[5] and Pascal Chanez[6]

[1]Department of Translational Medical Sciences and Center for Basic and Clinical Immunology Research (CISI), School of Medicine, University of Naples Federico II, WAO Center of Excellence, Naples, Italy. [2]Institute of Experimental Endocrinology and Oncology (IEOS), National Research Council, Naples, Italy. [3]Institute for Lung Health, NIHR Leicester Biomedical Research Centre, University of Leicester, Leicester, UK. [4]School of Medicine and Public Health, University of Newcastle, Callaghan, Australia. [5]Center for Inflammation Research, Laboratory of Immunoregulation and Mucosal Immunology, VIB Center for Inflammation Research, Ghent, Belgium. [6]Department of Respiratory Diseases, Aix-Marseille University, Marseille, France. [7]G. Varricchi and C.E. Brightling contributed equally.

Corresponding author: Christopher E. Brightling (ceb17@leicester.ac.uk)

Check for updates

Shareable abstract (@ERSpublications)
Our growing understanding of how the epithelium and epithelial cytokines orchestrate airway remodelling in severe asthma is enabling the development of new therapies that may make disease remission a realistic treatment goal https://bit.ly/3UOblpT

Cite this article as: Varricchi G, Brightling CE, Grainge C, *et al*. Airway remodelling in asthma and the epithelium: on the edge of a new era. *Eur Respir J* 2024; 63: 2301619 [DOI: 10.1183/13993003.01619-2023].

This extracted version can be shared freely online.

Copyright ©The authors 2024.

This version is distributed under the terms of the Creative Commons Attribution Licence 4.0.

Received: 22 Sept 2023
Accepted: 15 Feb 2024

Abstract

Asthma is a chronic, heterogeneous disease of the airways, often characterised by structural changes known collectively as airway remodelling. In response to environmental insults, including pathogens, allergens and pollutants, the epithelium can initiate remodelling *via* an inflammatory cascade involving a variety of mediators that have downstream effects on both structural and immune cells. These mediators include the epithelial cytokines thymic stromal lymphopoietin, interleukin (IL)-33 and IL-25, which facilitate airway remodelling through cross-talk between epithelial cells and fibroblasts, and between mast cells and airway smooth muscle cells, as well as through signalling with immune cells such as macrophages. The epithelium can also initiate airway remodelling independently of inflammation in response to the mechanical stress present during bronchoconstriction. Furthermore, genetic and epigenetic alterations to epithelial components are believed to influence remodelling. Here, we review recent advances in our understanding of the roles of the epithelium and epithelial cytokines in driving airway remodelling, facilitated by developments in genetic sequencing and imaging techniques. We also explore how new and existing therapeutics that target the epithelium and epithelial cytokines could modify airway remodelling.
